# Higher Prevalence of Non-thyroidal-Illness Syndrome in Elderly Male Patients With Active *Helicobacter pylori* Infection

**DOI:** 10.3389/fmed.2021.682116

**Published:** 2021-07-08

**Authors:** Banruo Sun, Xuanping Wang, Michael Edmund David McLarnon, Yu Ding, Miao Liu, Wei Dai, Gangshi Wang

**Affiliations:** ^1^Department of Endocrinology, Second Medical Center, Chinese People's Liberation Army General Hospital, National Clinical Research Center for Geriatric Diseases, Beijing, China; ^2^School of Medicine, Dentistry and Biomedical Sciences, Queen's University Belfast, Belfast, United Kingdom; ^3^Department of Gastroenterology, Second Medical Center, Chinese People's Liberation Army General Hospital, National Clinical Research Center for Geriatric Diseases, Beijing, China; ^4^Department of Statistics and Epidemiology, Graduate School of Chinese People's Liberation Army General Hospital, Beijing, China; ^5^Office of Information Management, Second Medical Center, Chinese People's Liberation Army General Hospital, Beijing, China

**Keywords:** *Helicobacter pylori*, non-thyroidal-illness syndrome, thyroid function, thyroid morphology, elderly, male

## Abstract

**Objective:** It is currently unclear whether the *Helicobacter pylori (H. pylori)* infection leads to associated alterations in thyroid functions and thyroidal illnesses. This study aims to analyse this relationship in an elderly male cohort over a five-year period.

**Design:** A case retrospective study.

**Methods:** A longitudinal study was designed to collect subjects (≥65 years old) receiving both a thyroid examination and *H. pylori* infection status determined by ^13^C-urea breath test in 2013 at our unit. Subjects were followed every 1 to 2 years until December 2017 for laboratory results, visits to outpatient clinics/emergency departments etc. Blood tests and thyroid ultrasonography were performed to determine thyroid function and morphology.

**Results:** 356 male subjects with mean age 78.5 ± 9.8 years were included. Active *H. pylori* infection was positive in 88 subjects (24.7%). Thyroid function tests and ultrasonography showed similar patterns between *H. pylori* positive and negative groups. Non-thyroidal-illness syndrome (NTIS) was diagnosed in 30/210 (14%) patients who experienced acute illnesses and hospitalization over five-year follow-up. Notably, NTIS demonstrated significantly higher prevalence in the *H. pylori* positive group compared to the negative group (17.1 vs. 5.6%, *P* = 0.001). Multivariate analysis showed that when age, APACHE II score and hemoglobin levels were adjusted, *H. pylori* status still has significant interrelationship with NTIS (OR = 3.497, *P* = 0.003).

**Conclusions:** There is a positive association between chronic active *H. pylori* infection and NTIS prevalence in this elderly male cohort. Further studies are needed to elucidate the role of *H. pylori* infection on NTIS in elderly male patients.

## Introduction

*Helicobacter pylori (H. pylori)* infection is a common disease, affecting nearly half the world population, with more than an 80% prevalence in some areas of Asia (1). Besides its harmful effects on the gastrointestinal system, *H. pylori's* impact on other organ systems has been studied, and can lead to coronary artery disease, metabolic syndrome and insulin resistance, type 2 diabetes mellitus, among many other illnesses (2). Out of these manifestations, the link between *H. pylori* infection and thyroid abnormalities [including autoimmune thyroid diseases (ATD)] has been researched (3), however, all of these have applied no restrictions on the age of participants.

As age increases, the possibility of thyroid function disturbances rises due to morphological and physiological changes of the thyroid gland, which may ultimately lead to both the hospitalization and death of the patient (4), demonstrating the importance of thyroid function in elderly patients' health status. Transcriptome analyses have identified changes in the “aging thyroid's” gene expression, such as mitochondrial dysfunction and the loss of proteostasis (5). International guidelines have specified treatment considerations for clinical and subclinical thyroid dysfunction in elderly people (6).

Similar to their thyroid status, elderly people are likely to be colonized for longer with *H. pylori* compared to younger patients, and they also are more likely to experience prominent and severe immune-sequelae from its infection. Persistent active inflammation induced by *H. pylori* infection can lead to autoimmune immunopathological reactivity in affected patients, involving almost all body compartments (7). Systemic immune and inflammatory responses to *H. pylori* have been found to be related to extra-gastrointestinal system diseases, including thyroid abnormalities (8), such as lower free thyroxine level, thyroid nodules, high thyroid volume, and ATD (9–11).

This paper is a retrospective longitudinal study analyzing the relationship between active *H. pylori* infection and the patients' thyroid function over a five-year period, with an exclusive focus on elderly patients. Thyroid status was examined by thyroid function test, thyroid antibody testing, and ultrasonography of the gland. We presented significant associations between *H. pylori* infection and non-thyroidal-illness syndrome (NTIS) in our cohort. NTIS is often present in the critically ill patient, affecting the patient's recovery and prognosis (12, 13); therefore it is worthwhile to investigate its clinical significance in an elderly cohort.

## Subjects and Methods

### Study Design

We identified subjects who had received both thyroid examination and ^13^C-urea breath test (^13^C-UBT) (which was used for active *H. pylori* detection), between January 2013 and December 2017 from the database of Second Medical Center, Chinese PLA General Hospital, Beijing. Participants aged 65 years or older were enrolled during their visits to outpatient clinics in 2013, with all essential clinical information being recorded, including patient demographics, diagnoses, their main underlying diseases, laboratory results, procedures, and prescriptions. The subjects were then followed up every 1 to 2 years until 2017, for laboratory results; hospitalization; visits to outpatient clinics and emergency departments; diagnoses; procedures; prescriptions; and death. Blood test and thyroid ultrasonography were performed on the same day or no longer than 1 week before or after the ^13^C-UBT. At least three ^13^C-UBT tests were performed for each subject, with one test at the beginning and one at the end of the follow-up. Patient comorbidities were collected and analyzed by the Charlson Comorbidity Index (CCI) scoring system (14). Clinical information from the patients who were hospitalized and/or visited the emergency department during the study period was additionally collected. The Acute Physiology and Chronic Health Evaluation II (APACHE II) score, widely used by Intensive Care Units to predict mortality (15), and Mini Nutritional Assessment Short-Form (MNA-SF) score, the screening tool for older patients' nutritional status (16), were recorded and analyzed in the first 24 h after admission. With regards to APACHE II score, it was sub-divided into groups scoring < 10 and ≥ 10, whereas for MNA-SF it was sub-categorized into scores < 12 and ≥ 12 groups.

At the start of study, we excluded subjects who had a diagnosis of hyperthyroidism or hypothyroidism, abnormal thyroid function, a prior history of thyroidal, hypophyseal or hypothalamic diseases, and those who were taking anti-thyroid or thyroid replacement medication and/or iodine-containing drugs. Subjects who had a conversion of ^13^C-UBT results (positive/negative alteration) and who became deceased during follow-up were also excluded. Furthermore, subjects taking drugs that might influence the results of ^13^C-UBT examination up to 4 weeks prior to screening (17) were additionally excluded from this study.

The study protocol was approved by the Institutional Review Board of Chinese PLA General Hospital (reference no: S2020-330-01). Since this is a retrospective statistical analysis on clinical data without disclosing the patients' identity, the need of consent was waived by the institutional ethical committee of the hospital.

### Laboratory Assessments

Thyroid function tests were assessed *via* measurements of thyroid stimulating hormone (TSH) and total T3 (TT3), free T3 (FT3), total T4 (TT4), free T4 (FT4), along with anti-thyroid peroxidase (anti-TPO) and anti-thyroglobulin (anti-Tg) using immunochemiluminescent assays (Cobas e601, Roche Diagnostics Ltd., Switzerland) with the following normal reference ranges: TSH (0.35–5.5 mU/ml); TT3 (1.01–2.95 nmol/L); FT3 (2.76–6.3 pmol/L); TT4 (55.34–160.88 nmol/L); FT4 (10.42–24.32 pmol/L); anti-TPO (<60 IU/ml); anti-Tg (<60 IU/ml). Blood counts and blood chemistry panels were determined using an automated electronic counter (Sysmex XN3000, Sysmex Corporation, Kobe, Japan) and chemiluminescence on an autoanalyzer (Cobas c501, Roche Diagnostics Ltd., Switzerland), respectively.

### Measurements

*H. pylori* infection was detected *via* a ^13^C-breath test instrument (Fischer Analysen Instrumente GmbH, Germany). The results of ^13^C-UBT were reported as *H. pylori* positive (delta over baseline value ≥ 4) or negative (delta over baseline value < 4). Thyroid nodules were determined by ultrasonography, and both the longest diameter and maximum cross-sectional area were calculated according to patient medical records. Enlargement of thyroid nodules was defined as the length and width of nodules increasing by more than 20%. The criteria for NTIS (18) applied in our study were as follows: (1) TT3 level <1.01 nmol/L and/or FT3 level <6.3 pmol/L; (2) TSH at the upper normal limit of 5.5 mU/L; (3) TT3 and FT3 level returned to normal range when the patients' condition improved or when they were discharged from hospital. Anemia was defined as hemoglobin values lower than 130.0 g/L (19).

### Statistical Analysis

The Kolmogorov–Smirnov test was used to assess normal distribution variables. Student's *t*-test and analysis of variance (ANOVA) were used to compare continuous variables. One-way ANOVA and simple linear correlation were used to assess the relationship between continuous variables. Logistic regression was used to assess the relative influence of independent variables on *H. pylori* infection and calculate the odds ratios (ORs) and 95% confidence intervals (CIs). Continuous variables are presented as mean ± standard deviation (SD) and median (interquartile range) for variables of skewness distribution. Categorical variables were expressed as *n* (%). A P value of <0.05 was considered significant. Statistical analysis was performed using Statistical Package for SPSS (Windows version 19.0).

## Results

### Participants' General Information and Investigations

**Figure 1:**
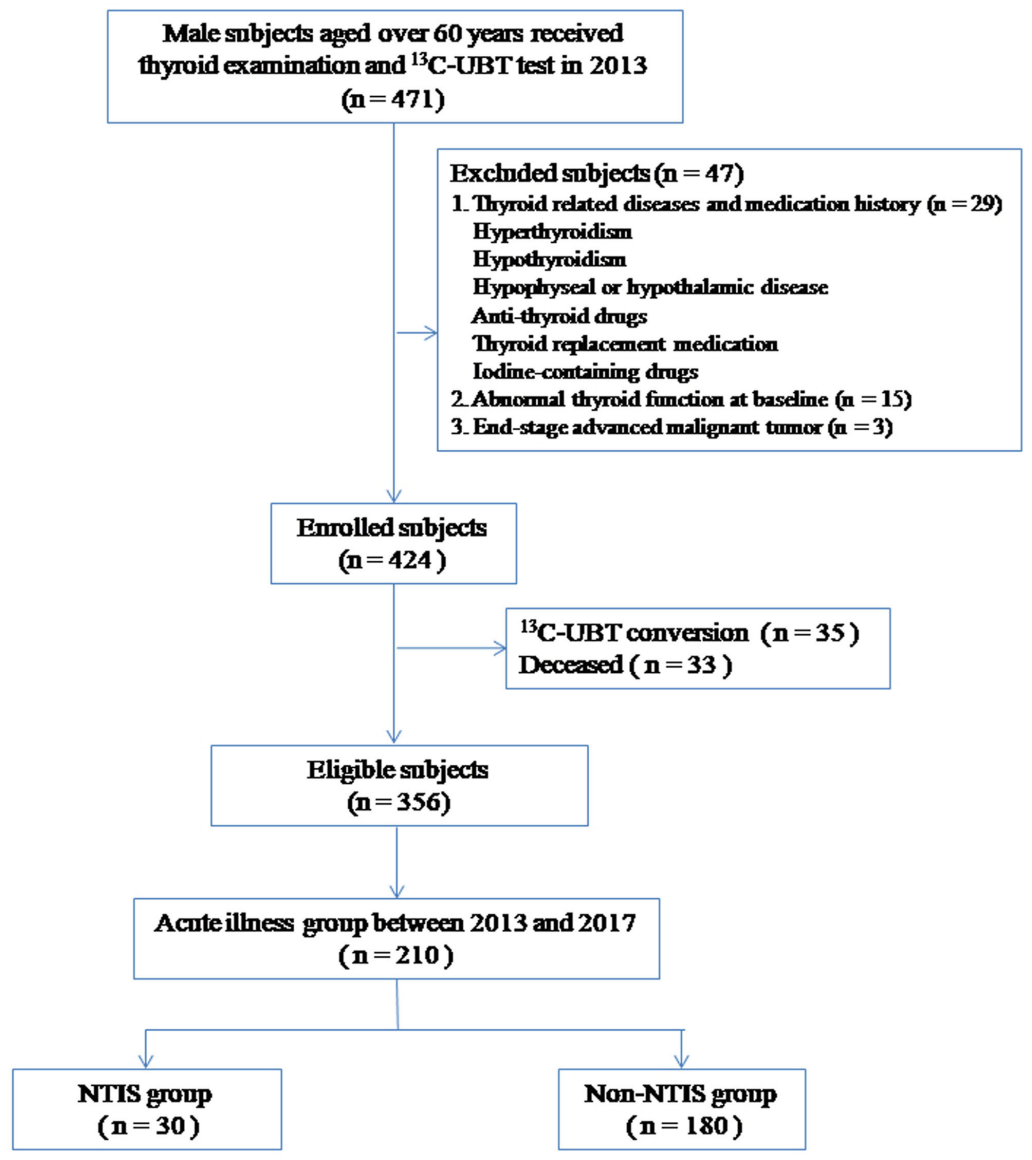
Participant's recruitment flow diagram.

At the beginning of the study, 471 male subjects aged over 65 years were grouped. 47 were excluded according to the exclusion criteria, 33 were deceased and 35 showed ^13^C-UBT conversion during the follow-up. Finally, a total of 356 subjects were enrolled in the analysis (Figure 1). The mean age was 78.5 ± 9.8 years.

*H. pylori* positive results were present in 88 subjects (24.7%). A significantly higher portion of *H. pylori* positive patients were allocated in the ≥80 year group (61.4%) which demonstrated a correlation with patient age (*P* = 0.037). Patient comorbidities were measured *via* the CCI score, in which 65.4% of the participants (233 patients) scored < 3 points and 34.6% (123 patients) scored ≥ 3 points, but no interrelationship was found between *H. pylori* and particular CCI ranges.

The hemoglobin level was relatively lower in the *H. pylori* positive group compared to the negative group (137.3 ± 15.2 vs. 140.6 ± 14.5 g/L, *P* = 0.062). Eighty-one cases with hemoglobin <130 g/L were observed in this cohort. In general, the prevalence of anemia in the *H. pylori* positive group was higher than that in the negative group (30.7 vs. 20.1%, *P* = 0.041). There were no differences in the liver function tests, renal function tests, blood lipids, and chemical elements among the two groups (Table 1).

**Table 1:** Demographic information of participants at the beginning of the study.

**Characteristics**	**Study population** **(*n* = 356)**	***H. pylori*** **status**		***P*-value**
		**Negative (*n* = 268)**	**Positive (*n* = 88)**	
**Mean ± SD**				
Age (years)	78.5 ± 9.8	77.9 ± 9.7	80.1 ± 10.0	0.081
Hemoglobin (g/L)	139.7 ±14.7	140.6 ± 14.5	137.2 ± 15.3	0.062
Serum albumin (g/L)	45.6 ± 2.8	45.7 ± 2.9	45.3 ± 2.4	0.175
Alanine aminotransferase (U/L)	18.0 ± 8.2	17.6 ± 7.7	19.4 ± 9.4	0.101
Serum creatinine (μmol/L)	87.4 ± 19.2	87.5 ± 19.2	87.1± 19.3	0.818
Glomerular filtration rate (ml/min/1.73m^2^)	82.4 ± 20.3	82.6 ± 21.1	82.0 ± 17.9	0.269
Serum uric acid (μmol/L)	353.9 ± 77.2	357.6 ± 76.1	342.8 ± 80.2	0.119
Glycated hemoglobin A1c (%)	6.11 ± 0.61	6.10 ± 0.61	6.20 ± 0.61	0.490
Total cholesterol (mmol/L)	4.49 ± 0.96	4.44 ± 0.95	4.64 ± 0.97	0.094
Triglyceride (mmol/L)	1.39 ± 0.67	1.36 ± 0.62	1.48 ± 0.79	0.120
Low density lipoprotein-c (mmol/L)	2.72 ± 0.83	2.68 ± 0.82	2.82 ± 0.85	0.141
High density lipoprotein-c (mmol/L)	1.35 ± 0.35	1.35 ± 0.34	1.34 ± 0.39	0.790
***N*** **(%)**				
**Age (years)**				
<80	173 (48.6%)	139 (51.9%)	34 (38.6%)	0.037
≥80	183 (51.4%)	129 (49.1%)	54 (61.4%)	
**CCI score**				
<3	233 (65.4%)	180 (67.2%)	53 (60.2%)	0.247
≥3	123 (34.6%)	88 (32.8%)	35 (39.8%)	
**Anemia**				
Positive	81 (22.8%)	54 (20.1%)	27 (30.7%)	0.041
Negative	275 (77.2%)	214 (79.9%)	61 (69.3%)	

### Thyroid Investigations at the Beginning of the Study

TT3, TT4, FT3, and FT4 levels did not differ significantly between the *H. pylori* positive and negative groups upon enrollment in this study. TSH levels were similar between these groups, as was the T3/T4 ratio and the presence of thyroid antibodies (anti-TPO and anti-Tg). The features of thyroid ultrasonography results (as shown in Table 2) were similarly distributed between these two groups.

**Table 2:** Results of thyroid tests of participants.

	**Study population** **(*n* = 356)**	***H. pylori*** **status**		***P-*value**
		**Negative (*n* = 268)**	**Positive (*n* = 88)**	
**Mean ± SD**				
TT3 (nmol/L)	1.50 ± 0.32	1.51 ± 0.31	1.48 ± 0.36	0.501
TT4 (nmol/L)	97.0 ± 21.9	96.8 ± 21.3	97.9 ± 23.6	0.688
FT3 (pmol/L)	4.30 ± 0.75	4.32 ± 0.72	4.25 ± 0.83	0.469
FT4 (pmol/L)	15.9 ± 2.5	16.0 ± 2.41	15.8 ± 2.60	0.529
TSH (mU/L)	2.43 ± 1.24	2.46 ± 1.24	2.34 ± 1.23	0.435
T3/T4 (%)	1.59 ± 0.31	1.60 ± 0.31	1.60 ± 0.32	0.212
***N*** **(%)**				
**Thyroid antibodies**				
Anti-TPO positive	18 (5.1%)	16 (6.0%)	2 (2.3%)	0.261
Anti-Tg positive	35 (9.8%)	29 (10.8%)	6 (6.8%)	0.310
Anti-TPO and/or Anti-Tg positive	37 (10.4%)	31 (11.6%)	6 (6.8%)	0.205
**Ultrasonography**				
Normal	86 (24.2%)	65 (24.3%)	21 (23.9%)	0.941
Thyroiditis	7 (2.0%)	4 (1.5%)	3 (3.4%)	0.261
Thyroid nodule	263 (73.9%)	199 (74.3%)	64 (72.7%)	0.777
Thyroiditis with nodule	6 (1.7%)	5 (1.9%)	1 (1.1%)	0.645
Multinodular goiter	53 (14.9%)	36 (13.4%)	17 (19.3%)	0.178
Uninodular goiter	204 (57.2%)	158 (58.9%)	46 (52.3%)	0.272
**Median (interquartile range)**				
**Thyroid nodule**				
Longest diameter	0.7 (0.4-1.05)	0.6 (0.4-1.0)	0.7 (0.4-1.1)	0.940
Maximum cross sectional area	0.3 (0.12-0.77)	0.3 (0.12-0.77)	0.26 (0.1-0.86)	0.469

### Thyroid Investigations After 5 Years of Follow-Up

All 356 patients were followed up during the 5-year period, and they were still categorized based on their *H. pylori* status when they went through thyroid investigations. Overall, thyroid antibodies turned positive in 1.1% of all subjects, new thyroid nodules appeared in 30.1%, whereas nodule enlargement occurred in 32.3% of participants, primary hypothyroidism arose in 11.8% and primary hyperthyroidism emerged in 1.1% of the participants within the study group. Yet, none of these conditions' prevalence varies greatly between *H. pylori* positive and negative groups (Table 3 and Supplementary Table 1).

**Table 3:** Thyroid functions after 5 years of follow-ups.

	**Total** **(*n* = 356)**	***H. pylori*** **status**		***P*-value**
		**Negative (*n* = 268)**	**Positive (*n* = 88)**	
***N*** **(%)**				
**Thyroid dysfunction**				
Primary hypothyroidism	42 (11.8%)	33 (12.3%)	9 (10.2%)	0.599
Primary hyperthyroidism	4 (1.1%)	2 (0.8%)	2 (2.3%)	0.239
Antibody positive conversion	4 (1.1%)	3 (1.1%)	1 (1.1%)	0.990
**Thyroid nodule**				
New detected	28 (30.1%)	22 (31.9%)	6 (25.0%)	0.821
Enlargement	85 (32.3%)	70 (35.2%)	15 (23.4%)	0.092
NITS	30 (8.4%)	15 (5.6%)	15 (17.1%)	0.001

NTIS was newly diagnosed in 30 out of 210 patients who experienced acute illnesses and hospitalization during the 5-year follow-up. Among the 30 NTIS patients, the most diagnosis of acute illness upon hospitalization was pneumonia, followed by acute cerebral infarction and acute coronary syndrome. Detailed information was listed in Supplementary Table 2. No patient was hospitalized due to gastrointestinal bleeding or acute anemization due to peptic ulcer or gastric cancer. NTIS has demonstrated a significantly higher prevalence in the *H. pylori* positive group, for which the figure was approximately three times greater in comparison to the *H. pylori* negative group (17.1 vs. 5.6%, *P* = 0.001) (Table 3).

### Factors Correlate With NTIS

Our results have illustrated the positive correlation between *H. pylori* and NTIS (OR = 4.143, *P* = 0.001), however, multiple additional factors could also affect the prevalence of NTIS. Patients with NTIS have displayed a higher mean age compared to their non-NTIS counterparts, which were 85.0 ± 6.8 years and 81.9 ± 8.9 years accordingly (*P* = 0.033). Hemoglobin levels were significantly lower in the NTIS group which was 125.8 ± 16.7 g/L compared to 137.8 ± 13.9 g/L in the non-NTIS group (*p* = 0.001). The NTIS prevalence in anemia group was higher than that in the non-anemia group (25.8 vs. 9.0%, *P* = 0.001). In contrast, patients' CCI score, liver function, renal function, blood lipid, and thyroid function upon enrollment showed no correlation with the presence of NTIS. Neither the APACHE II nor the MNA-SF sub-groups demonstrated significant difference in their prevalence of NTIS. However, the NTIS proportion displayed higher values in the APACHE II score ≥ 10 group with a borderline *p*-value (20.0 vs. 10.8%, *P* = 0.063) (Supplementary Table 3). Multivariate analysis showed that when age, APACHE II score and anemia were adjusted, the *H. pylori* status still had significant correlation with NTIS (OR = 3.497, *P* = 0.003) (Table 4). We also studied the relationship between the occurrence of NTIS and patients' acute presenting diseases, including cardiovascular diseases, pulmonary diseases, stroke, trauma, etc. None of these diseases have demonstrated any significant relationship with the development of NTIS (*P* = 0.915, Supplementary Table 4).

**Table 4:** ORs and 95% CI of *H. pylori* positive on NTIS prevalence.

**Variable**	**OR**	**95% CI**	***P*-value**
**Hospitalized patients (*****n*** **= 210)**			
Crude	4.143	1.852-9.269	0.001
Age adjusted	3.888	1.724-8.771	0.001
APACHE II adjusted	4.095	1.816-9.237	0.001
Anemia adjusted	3.619	1.583-8.277	0.002
Age and APACHE II adjusted	3.948	1.736-8.975	0.001
Age and anemia adjusted	3.481	1.513-8.007	0.003
APACHE II and anemia adjusted	3.578	1.553-8.246	0.003
Age, APACHEII and anemia adjusted	3.497	1.510-8.100	0.003

## Discussion

The most important finding from this study is that *H. pylori* infection over a chronic time frame exhibits significant correlation to NTIS prevalence in an elderly male cohort, and to the best of our knowledge this is the first study reporting this association.

NTIS is a term used to encompass a syndrome whereby there is an alteration in thyroid hormone function during non-thyroidal illness (20); characteristically low T3 and/or T4 levels and increased reverse T3 levels (and in some cases reduced or normal TSH) on thyroid hormone profile, despite the patient being clinically euthyroid (21, 22). It has been well-documented in critically-ill patients (23), but it is also generally accepted that it can occur as an adaptive response secondary to nearly any type of acute or chronic illness (21). This is thought to result from an allostatic response from the thyroid in response to conditions of strain or stress, possibly to conserve energy (24). Clinically, there may be no symptoms or signs of NTIS in an affected patient, and indeed there are no findings specific to the condition (25). Some debates exist as to whether the illness requires medical management, in the form of hormone replacement (25, 26), but definitive management lies in treating the underlying disease contributing to systemic inflammation. NTIS is very common in critically-ill, hospitalized elderly patients, emerging as the most sensitive independent predictor of short-term survival. Research indicates that the NTIS prevalence (specifically low T3 syndrome) was 31.9% in hospitalized elderly patients. The mortality rate was significantly higher among patients with low T3 syndrome, which emerged as the sole predictive factor of death (27).

It is plausible that the inflammatory processes related to chronic *H. pylori* infection may have systemic manifestations, and this could lead to an adaptive response such as that seen in NTIS. Indeed, there are studies demonstrating that chronic *H. pylori* infection may extend to inflame and compromise extra-intestinal organs; in particular, endocrine organs (28). Since NTIS may develop on a background of any chronic inflammatory illness, it seems plausible that *H. pylori* may also be contributing to NTIS in our patient cohort through similar means.

Although there has been no direct evidence suggesting the relationship between *H. pylori* and NTIS to date, a study investigating the link between NTIS and chronic obstructive pulmonary disease (COPD) found that tumor necrosis factor-α (TNF-α) and interleukin-6 (IL-6) were significantly higher in NTIS patients (29). Meanwhile, *H. pylori*-mediated chronic inflammation has also been shown to increase the expression of TNF-α and IL-6 (30, 31). A causal role for IL-6 in the development of NTIS in mice has been shown since IL-6 knock out mice show a less pronounced drop in serum T3 during illness (32). Infusions of rTNF in men appears to decrease serum T3 and TSH and increase reverse-T3 (rT3) (33), leading to the thyroid hormone profile characteristically seen in NTIS.

Under acute illnesses, patients experience the same NTIS hormonal pattern *via* the manipulation type 2 deiodinase (D2) and type 1 deiodinase (D1), with the subsequent activation of cytokines TNF, IL-1, IL-6, and NF-kB (12), which may exhibit some common molecular pathways irrespective of the underlying disease in question. In our study, a similar likelihood of NTIS was found in different disease types; this demonstrates that NTIS prevalence was independent to the disease category. Our findings are further backed up by Plikat et al. (34), who has stated that the frequency of NTIS is only influenced by disease severity and prognosis, rather than disease type.

However, aforementioned in our results, there were additional factors identified possibly correlating with the development of NTIS other than *H. pylori*, and in our study we have demonstrated a potential link between APACHE II score and NTIS (*P* = 0.063, borderline significant). APACHE II score is used for disease severity classification typically for Intensive Care Unit patients (15), and a higher score is indicative of greater severity of systemic illness and a subsequently higher mortality rate. Multiple pieces of evidence have shown that NTIS emergence is closely related to the severity of illness of the host (12); this is due to the upregulation of type 3 deiodinase (D3) in severely-ill patients, which inactivates both T4 and T3 molecules and creates rT3, as well as increasing D2 expression, which activates macrophages and cytokines, resulting in NTIS patterns via hypothalamic pituitary axis interference and thyroid hormones binding inhibition (25). Therefore, a potential link can be established between high APACHE II score and the development of NTIS.

We have also demonstrated a significant association between NTIS and baseline serum hemoglobin (*P* < 0.05) suggesting anemia itself may contribute to the development of NTIS. Anemia due to chronic illness is often referred to as anemia of chronic disease (also known as anemia of inflammation) (35). In this context, anemia is evidence of an activated immune system and has been proposed to be the result of a protective defense strategy (36); this parallels to NTIS which is thought to be an allostatic response (24). A significant association (*P* = 0.041) between active *H. pylori* infection and anemia was identified at the beginning of the study, which corroborated our previous findings (37). While there is an inverse relationship between TNF-α and hemoglobin (38), this also adds consideration to the hypothesis that inflammation in *H. pylori* may cause the initial rise of TNF-α, which itself may potentially lead to anemia. As anemia is a marker of inflammation that may result as part of an adaptive response as is the case in NTIS, it is possible to establish a plausible link between *H. pylori*, anemia and NTIS. Our study worked within the hypothesis that anemia was secondary to inflammation, but it should also be kept in mind that individuals experienced anemia secondary to acute or chronic gastrointestinal bleeding. In the clinical practice, acute gastrointestinal bleeding is a common entity in the elderly due to peptic ulcer or gastric cancer which is closely related to *H. pylori* infection. Furthermore, it is common for elderly patients to take long term aspirin and other anticoagulants, and we are also aware of the effects of these drugs on gastric bleeding, when the incidence was even higher with the coexistence of *H. pylori* (39). However, no gastrointestinal bleeding case was exhibited in the NTIS group in this study, we did not focus on drugs such as aspirin and anticoagulants in this group. The only cancerous patient in the NTIS group (Supplementary Table 2) was hospitalized due to obstruction of the gastric cardia, and his hemoglobin was normal and fecal occult blood test was negative when the NTIS occured. All in all, it could be speculated that eradication of *H. pylori* would help to reduce the prevalence of NTIS subsequent to acute gastrointestinal bleeding in the elderly.

In our cohort, we additionally showed no association between *H. pylori* infection and ATD. The relationship between *H. pylori* and ATD is an area of some uncertainty, with some studies finding positive associations whereas others find no correlation (28). However, a lack of information regarding this association in Asian populations has been previously identified (3) and our results add to the literature in this area. Furthermore, even within the continent, *H. pylori* prevalence is shown to differ widely by geography (40), as does genotypic variation (41), and our results at least suggest no correlation between *H. pylori* infection and ATD in our study cohort. Although NTIS is a disturbance in thyroid function, the causative agent is the underlying chronic inflammation secondary to another illness (21). Owing to the high prevalence of chronic *H. pylori* infection in elderly people, it could be speculated that *H. pylori* infection in combination with physiological changes of aging process could lower the NTIS threshold and increase its occurrence in this specific population, although no causal relationship could be found between *H. pylori* infection and NTIS yet. Therefore, clinically ensuring that *H. pylori* is identified and eradicated in individuals colonized with the bacteria may help to reduce subsequent development of NTIS, which in turn could improve patient prognosis, especially in elderly patients.

Our study has several limitations. Although ^13^C-UBT has the greatest combination of sensitivity and specificity (41) for *H. pylori* infection diagnosis and the guideline recommends it as the first choice of non-invasive test in the clinical practice, especially in the elderly (42), false positive or negative ^13^C-breath test results should be noticed since they are correlated with gastric atrophy, common in the elderly (43). Therefore, using additional confirmatory testing (i.e., biopsy for histological diagnosis) has the potential to verify the validity of *H. pylori* diagnosis. As a retrospective study from a single center, rT3 data was not available to be recorded from our data collection. Nevertheless, serum concentrations of rT3 can be increased or remain unchanged in both acute and chronically critically ill patients with NTIS (24). Furthermore, *H. pylori* strain and genotypic markers are not reported, which may relate to disease risk and clinical outcome (44). We noticed that all our samples are males and cannot be representative of the whole population, however, we have not excluded any patients base on their sex and gender. Unfortunately, we could enroll only male subjects due to the nature of our institution. We believe this elderly-male sample has also helped to exclude the impact of gender on our results. Conversely, a relatively large study population with equivalent gender distribution investigating multiple clinical outcomes over a long period of time, with multivariate analysis to identify potentially confounding factors, has the potential to add both validity and weight to our study. Additionally, our study only established indefinite correlations between variables due to its observational nature and would be more reliable if the underlying molecular mechanisms were researched further (i.e., detection of IL-6, TNF etc.), which could indicate the direction of future study. As the first study of its kind, further research is necessary in order to make clinical recommendations and conclusions on the association.

## Conclusion

There is a positive association between chronic active *H. pylori* infection and the prevalence of NTIS in this elderly male cohort over a 5-year period. The prevalence of NTIS tends to increase with age, low hemoglobin and high APACHE II score, but not with underlying illnesses. We found no correlation between *H. pylori* infection and ATD or thyroid morphological (ultrasonographic) investigation. Further studies are needed to delineate the role and underlying mechanisms of chronic *H. pylori* infection on NTIS in the elderly, then to provide valuable suggestions to geriatric practice.

## Data Availability Statement

The raw data supporting the conclusions of this article will be made available by the authors, without undue reservation.

## Ethics Statement

The studies involving human participants were reviewed and approved by The ethical committee of the Chinese PLA General Hospital, Beijing, China had permitted to use the data for this project. The patients/participants provided their written informed consent to participate in this study.

## Author Contributions

BS collected data, performed the data analysis, statistical analysis, and manuscript preparation. XW and MM searched literature, conducted research, and wrote the initial manuscript. YD and WD collected data and conducted research. ML performed statistical analysis. GW conceived and designed research, revised the manuscript, and had primary responsibility for final content. All authors read and approved the final manuscript.

## Conflict of Interest

The authors declare that the research was conducted in the absence of any commercial or financial relationships that could be construed as a potential conflict of interest.

## Abbreviations

^13^C-UBT: ^13^C-urea breath test
anti-Tg: anti-thyroglobulin
anti-TPO: anti-thyroid peroxidase
APACHE II: Acute Physiology and Chronic Health Evaluation II
ATD: autoimmune thyroid diseases
CCI: Charlson comorbidity index
CIs: Confidence intervals
COPD: Chronic obstructive pulmonary disease
CRP: C-reactive protein
D1: Type 1 deiodinase
D2: Type 2 deiodinase
D3: Type 3 deiodinase
DOB: Delta over baseline value of ^13^C-UBT
FT3: Free triiodothyronine
FT4: Free thyroxine
*H. pylori*: *Helicobacter pylori*
IL-6: Interleukin-6
MNA-SF: Mini Nutritional Assessment Short-Form
NF-κB: Nuclear factor-κ-gene binding
NTIS: Non-thyroidal-illness syndrome
OR: Odds ratio
rT3: Reverse-triiodothyronine
SD: Standard deviation
TFT: Thyroid function test
TNF-α: Tumor necrosis factor-α
TSH: Thyroid-stimulating hormone
TT3: Total triiodothyronine
TT4: Total thyroxine.
