# Supplementary material for: Higher Prevalence of Non-thyroidal-Illness Syndrome in Elderly Male Patients With Active Helicobacter pylori Infection
Source: Front Med (Lausanne). 2021 Jul 8;8:682116. doi: 10.3389/fmed.2021.682116 (PMC8295606; doi:10.3389/fmed.2021.682116)
Supplement: Supplementary file 1 [file Table_1.DOC]

**Supplementary table S1. Detailed results of thyroid investigations after 5 years of follow-up.**

|  | ***H. pylori* infection** | **5 years of follow-up** | | | | ***P* value** |
| --- | --- | --- | --- | --- | --- | --- |
|  |  | **Year 2013** | | **Year 2017** | |  |
| **TT3 (nmol/L)** | **+** | 1.52 ± 0.35 | 1.52 ± 0.26 | | | 0.980 |
|  | **-** | 1.54 ± 0.30 | 1.50 ± 0.36 | | | 0.237 |
| **TT4 (nmol/L)** | **+** | 99.4 ± 23.7 | 91.5 ± 17.3 | | | 0.044 |
|  | **-** | 96.7 ± 21.3 | 92.1± 18.5 | | | 0.021 |
| **FT3 (pmol/L)** | **+** | 4.40 ± 0.78 | 4.29 ± 0.54 | | | 0.406 |
|  | **-** | 4.38 ± 0.71 | 4.25 ± 0.69 | | | 0.047 |
| **FT4 (nmol/L)** | **+** | 15.9 ± 2.5 | 16.0 ± 2.8 | | | 0.813 |
|  | **-** | 15.9 ± 2.3 | 16.1 ± 2.6 | | | 0.368 |
| **TSH (mU/L)** | **+** | 2.45 ± 1.24 | 2.59 ± 1.49 | | | 0.540 |
|  | **-** | 2.45 ± 1.22 | 2.65 ± 1.68 | | | 0.667 |
| **T3/T4 (%)** | **+** | 1.56 ± 0.32 | 1.69 ± 0.27 | | | 0.011 |
|  | **-** | 1.63 ± 0.30 | 1.66 ± 0.37 | | | 0.280 |
| **Nodule growth Longest diameter (cm)** | **+** | 0.5 (0-0.8) | | 0.5 (0.3-0.8) | | 0.453 |
|  | **-** | 0.5 (0-0.9) | | 0.6 (0.3-1.0) | | 0.028 |
| **Maximum cross sectional area (cm2)** | **+** | 0.12 (0-0.42) | | 0.15 (0.03-0.48) | | 0.370 |
|  | **-** | 0.16 (0-0.54) | | | 0.24 (0.06-0.70) | 0.035 |

**Supplementary table S2.** **Data of NTIS patients**

| **Case No.** | **Clinical diagnosis** | ***H. pylori* status** | **Age (years)** | **Charlson score** | **APACHE II score** | **MNA-SF** | **TT4 ( nmol/L)** | **TT3 ( nmol/L)** | **FT3 ( pmol/L)** | **FT4 ( pmol/L)** | **TSH ( mU/mL)** |
| --- | --- | --- | --- | --- | --- | --- | --- | --- | --- | --- | --- |
| 1 | Renal pelvic carcinoma | Positive | 86 | 3 | 14 | 13 | 49.83 | 1.12 | 3.58 | 9.73 | 1.2 |
| 2 | Severe pneumonia | Positive | 90 | 2 | 20 | 10 | 63.68 | 0.851 | 1.84 | 14.96 | 0.888 |
| 3 | Acute cerebral infarction | Negative | 93 | 2 | 15 | 14 | 90.19 | 0.937 | 2.8 | 15.58 | 0.698 |
| 4 | Pneumonia | Positive | 87 | 5 | 12 | 12 | 117.2 | 0.66 | 1.78 | 18.24 | 2.09 |
| 5 | Kidney failure | Positive | 92 | 3 | 13 | 12 | 50.07 | 0.642 | 2.23 | 10.42 | 1.68 |
| 6 | Acute cerebral infarction | Positive | 87 | 5 | 12 | 14 | 43.44 | 0.76 | 3.23 | 11.79 | 0.964 |
| 7 | Acute coronary syndrome | Negative | 92 | 3 | 8 | 9 | 58.07 | 0.962 | 2.64 | 11.42 | 1.45 |
| 8 | Acute coronary syndrome | Negative | 91 | 3 | 12 | 12 | 75.17 | 0.912 | 2.59 | 16.17 | 1.33 |
| 9 | Acute coronary syndrome | Negative | 89 | 1 | 7 | 13 | 133.5 | 0.705 | 2.59 | 21.86 | 1.98 |
| 10 | Acute pancreatitis | Positive | 83 | 2 | 8 | 11 | 76.41 | 0.795 | 2.73 | 19.69 | 0.55 |
| 11 | Severe pneumonia | Negative | 88 | 5 | 11 | 11 | 112.2 | 0.909 | 3.09 | 25.49 | 0.929 |
| 12 | Lung cancer | Positive | 89 | 7 | 11 | 13 | 113.6 | 1.1 | 2.57 | 19.33 | 0.67 |
| 13 | Ischemic bowel disease | Positive | 85 | 3 | 8 | 13 | 66.47 | 0.764 | 3.86 | 12.23 | 1.51 |
| 14 | Pneumonia | Positive | 91 | 4 | 12 | 11 | 94.1 | 0.808 | 2.46 | 16.79 | 1.16 |
| 15 | Pneumonia | Positive | 92 | 2 | 8 | 11 | 109.3 | 0.957 | 3.28 | 20.86 | 3 |
| 16 | Femoral fracture | Negative | 86 | 2 | 15 | 14 | 103.8 | 0.825 | 2.58 | 22.59 | 2.16 |
| 17 | Kidney failure | Negative | 85 | 5 | 8 | 14 | 76.06 | 0.959 | 2.71 | 14.32 | 1.05 |
| 18 | Urinary system infection | Negative | 86 | 2 | 11 | 13 | 76.79 | 1.06 | 2.21 | 14.09 | 2.58 |
| 19 | Pneumonia | Negative | 83 | 2 | 6 | 13 | 56.18 | 0.845 | 3.47 | 17.03 | 1.33 |
| 20 | Severe pneumonia | Positive | 85 | 3 | 15 | 13 | 68.41 | 0.862 | 2.29 | 14.82 | 1.03 |
| 21 | Dermatomyositis | Negative | 83 | 4 | 7 | 11 | 63.01 | 0.776 | 2.89 | 16.8 | 1.08 |
| 22 | Non-hodgkin lymphoma | Positive | 89 | 3 | 12 | 14 | 84.9 | 0.675 | 2.25 | 13.07 | 3.97 |
| 23 | Acute cerebral infarction | Negative | 76 | 1 | 8 | 14 | 71.51 | 0.852 | 2.93 | 12.49 | 3.41 |
| 24 | Acute coronary syndrome | Positive | 78 | 1 | 8 | 13 | 68.58 | 1.01 | 2.4 | 12.11 | 2.21 |
| 25 | Acute cerebral infarction | Negative | 72 | 2 | 8 | 14 | 91 | 0.898 | 2.68 | 20.06 | 2.14 |
| 26 | Pneumonia | Negative | 87 | 0 | 6 | 13 | 90.49 | 0.849 | 2.69 | 21.87 | 0.748 |
| 27 | Pneumonia | Negative | 70 | 2 | 9 | 14 | 67.18 | 0.741 | 1.96 | 11.56 | 1.81 |
| 28 | Gastric carcinoma | Positive | 92 | 3 | 10 | 11 | 48.58 | 0.868 | 3.12 | 10.91 | 2.7 |
| 29 | Acute gastroenteritis | Negative | 70 | 1 | 7 | 12 | 93.59 | 0.898 | 2.76 | 19.31 | 3.27 |
| 30 | Acute cerebral infarction | Positive | 73 | 1 | 10 | 14 | 71.93 | 0.961 | 3.9 | 13.8 | 2.08 |

**Supplementary table S3: Potential factors correlate with NTIS.**

| **Characteristics** | **Numberof cases**  **(n=210 )** | **NTIS** | | | ***P* value** |
| --- | --- | --- | --- | --- | --- |
|  |  | **YES (n=30)** | | **NO (n=180)** |  |
| **Upon enrollment** |  |  | |  |  |
| **N (%)** |  |  | |  |  |
| **Age (years)** |  |  | |  |  |
| **< 80** | 64 | 6 (9.4%) | 58 (90.6%) | |  |
| **≥ 80** | 146 | 24 (16.4%) | 122 (83.6%) | | 0.178 |
| **CCI score** |  |  |  | |  |
| **< 3** | 112 | 15 (13.4%) | 97 (86.6%) | |  |
| **≥ 3** | 98 | 15 (15.3%) | 83(84.7%) | | 0.693 |
| ***H. pylori* status** |  |  |  | |  |
| **Positive** | 50 | 15 (30.0%) | 35 (70.0%) | |  |
| **Negative** | 160 | 15 (9.4%) | 145 (90.6%) | | 0.001 |
| **Hemoglobin (g/L)** |  |  |  | |  |
| **<130** | 66 | 17 (25.8%) | 49 (74.2%) | |  |
| **≥130** | 144 | 13 (9.0%) | 131 (91.0%) | | 0.001 |
| **Mean ± SD** |  |  |  | |  |
| **Age (years)** | 82.4 ± 8.7 | 85.0 ± 6.8 | 81.9 ± 8.9 | | 0.033 |
| **Hemoglobin (g/L)** | 136.1 ± 14.9 | 125.8 ± 16.7 | 137.8 ± 13.9 | | 0.001 |
| **Serum albumin (g/L)** | 45.3 ± 2.9 | 44.8 ± 2.7 | 45.4 ± 3.0 | | 0.297 |
| **Alanine aminotransferase (U/L)** | 18.2 ± 8.8 | 19.1 ± 12.0 | 18.0 ± 8.2 | | 0.531 |
| **Serum creatinine (μmol/L)** | 89.7 ± 21.0 | 92.7 ± 27.6 | 89.2 ± 19.7 | | 0.395 |
| **Glomerular filtration rate (ml/min/1.73m^2^)** | 79.9 ± 21.8 | 79.0 ± 25.4 | 80.1 ± 21.2 | | 0.807 |
| **Serum uric acid (μmol/L)** | 355.7 ± 80.9 | 348.5 ± 108.2 | 356.9 ± 75.8 | | 0.600 |
| **Hemoglobin A1c (%)** | 6.17 ± 0.66 | 6.31 ± 0.67 | 6.15 ± 0.66 | | 0.230 |
| **Total cholesterol (mmol/L)** | 4.48 ± 0.93 | 4.46 ± 1.06 | 4.49 ± 0.91 | | 0.868 |
| **Triglyceride (mmol/L)** | 1.40 ± 0.69 | 1.45 ± 0.97 | 1.39 ± 0.63 | | 0.837 |
| **Low density lipoprotein -c (mmol/L)** | 2.68 ± 0.84 | 2.58 ± 0.94 | 2.70 ± 0.82 | | 0.473 |
| **High density lipoprotein -c (mmol/L)** | 1.35 ± 0.38 | 1.43 ± 0.48 | 1.33 ± 0.36 | | 0.205 |
| **TT3 (nmol/L)** | 1.45 ± 0.29 | 1.40 ± 0.21 | 1.46 ± 0.31 | | 0.294 |
| **TT4 (nmol/L)** | 95.9 ± 22.2 | 93.0 ± 19.0 | 96.4 ± 22.6 | | 0.431 |
| **FT3 (pmol/L)** | 4.15 ± 0.63 | 4.05 ± 0.54 | 4.17 ± 0.64 | | 0.326 |
| **FT4 (pmol/L)** | 15.9 ± 2.5 | 15.6 ± 3.5 | 15.8 ± 2.4 | | 0.705 |
| **TSH (mU/L)** | 2.48 ± 1.23 | 2.30 ± 0.82 | 2.52 ± 1.28 | | 0.275 |
| **T3/T4 (%)** | 1.54 ± 0.29 | 1.47 ± 0.35 | 1.55 ± 0.28 | | 0.203 |
| **Upon NTIS** |  |  |  | |  |
| **N (%)** |  |  |  | |  |
| **APACHEⅡ score** |  |  |  | |  |
| **<10** | 130 | 14(10.8%) | 116(89.2%) | | 0.063 |
| **≥10** | 80 | 16(20.0%) | 64(80%) | |  |
| **MNA-SF score** |  |  |  | |  |
| **<12** | 46 | 8(17.4%) | 38(82.6%) | | 0.496 |
| **≥12** | 164 | 22(13.4%) | 142(86.6%) | |  |

**Supplementary table S4. Relation between underlying illnesses and NTIS**

| **Acute critical illnesses** | **NTIS** | | **Total** | **percentage** | ***P* value** |
| --- | --- | --- | --- | --- | --- |
|  | **Positive** | **Negative** |  |  |  |
| **Cardiovascular diseases** | 4 | 26 | 30 | 13.3 % | 0.915 |
| **Stroke** | 5 | 42 | 47 | 10.6 % |  |
| **Pneumonia or AECOPD** | 13 | 57 | 70 | 18.6 % |  |
| **Gastrointestinal diseases** | 3 | 19 | 22 | 13.6 % |  |
| **Urinary system diseases** | 1 | 6 | 7 | 14.3% |  |
| **Trauma and Fracture** | 1 | 12 | 13 | 7.7 % |  |
| **Complications of Malignant Tumor** | 3 | 18 | 21 | 14.3 % |  |
